# Phage Display Derived Monoclonal Antibodies: From Bench to Bedside

**DOI:** 10.3389/fimmu.2020.01986

**Published:** 2020-08-28

**Authors:** Mohamed A. Alfaleh, Hashem O. Alsaab, Ahmad Bakur Mahmoud, Almohanad A. Alkayyal, Martina L. Jones, Stephen M. Mahler, Anwar M. Hashem

**Affiliations:** ^1^Faculty of Pharmacy, King Abdulaziz University, Jeddah, Saudi Arabia; ^2^Vaccines and Immunotherapy Unit, King Fahd Medical Research Center, King Abdulaziz University, Jeddah, Saudi Arabia; ^3^Department of Pharmaceutics and Pharmaceutical Technology, College of Pharmacy, Taif University, Taif, Saudi Arabia; ^4^College of Applied Medical Sciences, Taibah University, Medina, Saudi Arabia; ^5^Department of Medical Laboratory Technology, University of Tabuk, Tabuk, Saudi Arabia; ^6^Australian Institute for Bioengineering and Nanotechnology, The University of Queensland, Brisbane, QLD, Australia; ^7^Australian Research Council Training Centre for Biopharmaceutical Innovation, The University of Queensland, Brisbane, QLD, Australia; ^8^Department of Medical Microbiology and Parasitology, Faculty of Medicine, King Abdulaziz University, Jeddah, Saudi Arabia

**Keywords:** monoclonal antibodies, phage display, antibody libraries, biopanning, biopharmaceuticals

## Abstract

Monoclonal antibodies (mAbs) have become one of the most important classes of biopharmaceutical products, and they continue to dominate the universe of biopharmaceutical markets in terms of approval and sales. They are the most profitable single product class, where they represent six of the top ten selling drugs. At the beginning of the 1990s, an *in vitro* antibody selection technology known as antibody phage display was developed by John McCafferty and Sir. Gregory Winter that enabled the discovery of human antibodies for diverse applications, particularly antibody-based drugs. They created combinatorial antibody libraries on filamentous phage to be utilized for generating antigen specific antibodies in a matter of weeks. Since then, more than 70 phage–derived antibodies entered clinical studies and 14 of them have been approved. These antibodies are indicated for cancer, and non-cancer medical conditions, such as inflammatory, optical, infectious, or immunological diseases. This review will illustrate the utility of phage display as a powerful platform for therapeutic antibodies discovery and describe in detail all the approved mAbs derived from phage display.

## Monoclonal Antibodies (mAbs)

**Figure 1:**
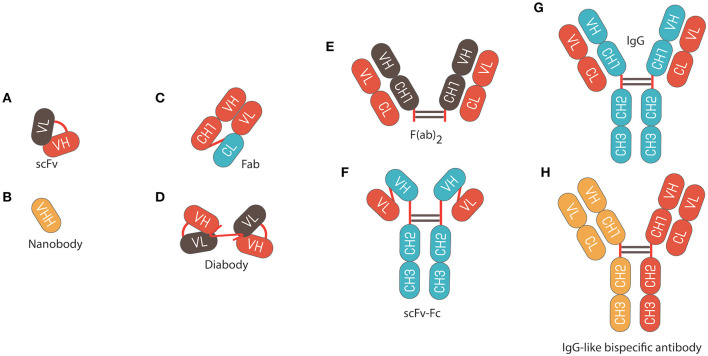
Schematic representation of different antibody formats. **(A)** Single chain fragment variable (scFv) composed of variable regions of the light chain (V_L_) linked to variable regions of the heavy chain (V_H_) by a flexible glycine-serine linker (Gly_4_Ser)_3_. **(B)** Nanobody fragments. **(C)** Fragment of antigen binding (Fab) composed of V_L_ and a constant domain of the light chain (CL) linked to V_H_ and constant domain 1 of the heavy chain (CH1) by a disulphide bond between the CL and CH1 domains. **(D)** Diabody composed of V_L_ linked to variable heavy V_H_ by a pentameric (Gly_4_Ser). **(E)** F(ab)2 fragment composed of 2 × Fab fragments joined by an Immunoglobulin G (IgG) hinge region. **(F)** scFv fusion with an Fc IgG. **(G)** IgG composed of constant fragment (Fc), which is able to bind and stimulate immune effector cells, and Fab, which comprises the variable domains that contain the antigen binding regions. **(H)** Bispecific IgG antibody.

Monoclonal antibodies (mAbs) are versatile biomacromolecules that can bind with high specificity to a wide range of protein and non-protein targets (1–4). These mAbs can be engineered and produced into different formats to enhance their functionality and use (Figure 1) (5). To date, more than 80 mAbs have been approved for clinical applications with many more under pre-clinical and clinical development (6). They represent six of the top ten selling drugs (7) with annual sales exceeding $120 billion in 2017 (8) and are expected to reach $130–200 billion by 2022 (9). They also have a high success rate in clinical development; for instance, it has been reported that the probability of FDA approval for mAbs in phase I of development is ~14.1%, which is almost twice the approval rate of small molecule drugs (~7.6%) (10, 11). Such factors make biopharmaceutical companies more motivated and willing to sponsor the development of these pharmaceutical products.

During the last 120 years, the research and development of antibody-related technologies have been the subject of four Nobel Prizes. In 1901, Emil von Behring won the first Nobel Prize in Physiology or Medicine for the successful therapeutic use of horse hyperimmune serum containing neutralizing polyclonal antibodies against diphtheria and tetanus toxins (12). Kohler and Milstein received the 1984 Nobel Prize in Physiology or Medicine for developing the ground-breaking hybridoma technology which facilitated the isolation of mAbs and their subsequent production in laboratories (13). In 2018, George P. Smith and Sir Gregory P. Winter were awarded with the Nobel Prize in Chemistry for their development of phage display of peptide and antibodies (14–16). In the same year, James P. Allison and Tasuku Honjo were honored by the 2018 Nobel Prize in Physiology or Medicine for their discoveries of cancer immunotherapy via the use of antibody blockade of the T-cell inhibitory receptor (CTLA-4) and programmed cell death protein 1 (PD1) to enhance anti-tumor immune responses (17, 18).

## Overview of Antibody Phage Display Libraries

Although hybridoma technology was ground-breaking at the time and still commonly used to produce antibodies as research reagents, murine-derived mAbs have limited therapeutic efficacy. Several reports have indicated that patients treated with murine-derived mAbs will develop a human anti-mouse antibody (HAMA) response, which accelerates mAb clearance, and could result in undesirable allergic reactions upon repeated administration (19, 20). Antibody engineering techniques have been subsequently utilized to create chimeric or humanized antibodies by utilizing the murine variable regions or complementary determining regions (CDRs), respectively, in conjunction with human constant regions, in order to maintain target specificity and reduce the HAMA response (21–23). Fully human antibodies are now generated using hybridoma technology in transgenic mice models, such as HuMabMouse and XenoMouse, whereby the mouse immunoglobulin (Ig) gene loci have been replaced with human loci within the transgenic mouse genome (24–26).

Development of antibody phage display libraries represents an alternative technique to the traditional hybridoma technology. They involve the isolation of fully human-derived mAbs from large Ig gene repertoires displayed on the surface of bacteriophages (16). In 1985, George P. Smith was the first to describe phage display technology by demonstrating that filamentous phages are able to display a peptide of interest on their surfaces after inserting a foreign DNA fragment into the filamentous phage coat protein gene (14). Subsequently, Parmley and Smith described a selection and affinity enrichment process known as “panning or biopanning,” that allowed for the isolation of peptide-phage fusions from a 10^8^-fold excess of wild type phages based on their specific binding affinity to biotinylated antibodies specific for the peptides (27). Later, McCafferty and Winter were the first to utilize phage display technology in antibody discovery by creating combinatorial antibody libraries on filamentous phages to be utilized for generating antigen specific mAbs (15, 16).

M13 is one of the filamentous bacteriophages (Ff) of *Escherichia coli* (*E. coli*), and one of the most widely used phages for antibody phage display (28, 29). Filamentous bacteriophages only infect *E. coli* strains through an interaction between the expressed F pilus on the surface of hosts, and a phage coat protein (30). M13 is a flexible cylindrical-shaped virus particle containing a circular single-stranded DNA genome (6,407-base) consisting of nine genes encoding for five coat proteins (pIII, pVIII, pVI, pVII, and pIX), and six assembly and replication proteins (31, 32). Most major phage display systems are based on pIII-antibody fusion proteins, due to pIII structural flexibility and its ability to display large proteins without losing its function (33–36).

**Figure 2:**
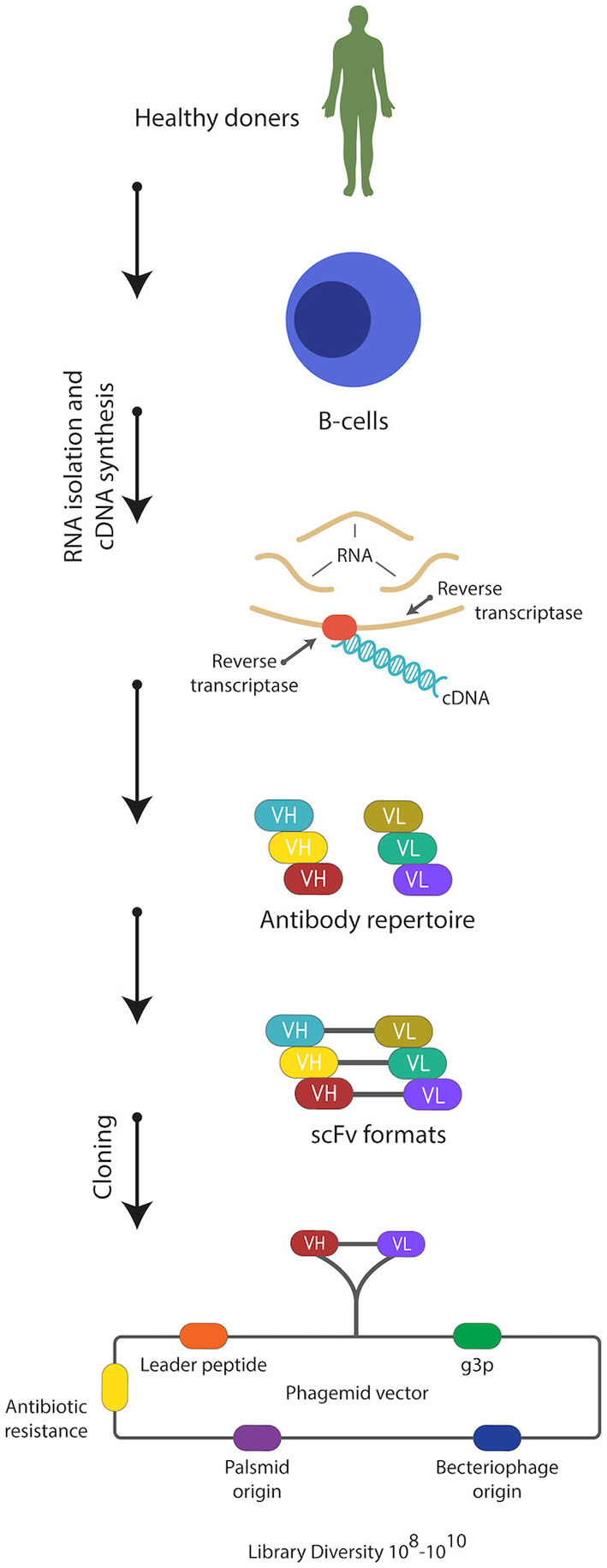
Strategy used for construction of naïve scFv-phage display libraries. Total RNA is isolated from B-lymphocytes from non-immunized healthy donors. Then cDNA is synthesized from the isolated RNA using reverse transcriptase enzyme. Then the repertoire of the V_H_ and V_L_ genes is amplified from the cDNA using forward and reverse primers hybridizing to the variable domains. scFvs are constructed and cloned into phagemid vector and a naïve phage library of 10^8^-10^10^ is usually generated.

The discovery of smaller recombinant antibody formats, such as variable domain [Fv; variable regions of the heavy (V_H_) or light chain (V_L_)], single-chain variable domain (scFv), diabodies (bivalent scFvs), heavy-domain camelid and shark antibody fragments (VHHs, nanobodies), and fragment antigen binding (Fab), has helped to advance antibody phage display technology (Figure 1) (37–42). These smaller fragments are more amenable to expression in bacteria compared to full antibodies, which require assembly of four polypeptide chains and extensive disulfide bond formation. For instance, creating a combinatorial scFv library on the surface of M13 filamentous phage has been achieved through combining populations of V_H_ and V_L_-domains, which are joined by a flexible, protease resistance glycine-serine linker (Gly_4_Ser)_3_, into a single DNA sequence (15). These antibody sequences are then introduced and cloned as a gene fusion with the bacteriophage pIII gene under the control of a weak promoter in a phagemid vector; a plasmid that carries an antibiotic resistance gene, bacterial and phage origins of replication (Figure 2) (43–46).

Co-infection of *E. coli* harboring a phagemid with a helper phage is essential for the formation of functional phage particles displaying pIII-antibody fusions (45). It causes *E. coli* to initiate the synthesis of all wild-type coat proteins needed for phage replication, and this is essential because the phagemid does not have all the genes necessary to encode a full bacteriophage in *E. coli* (47). The most commonly used helper phage is M13KO7, which is a derivative of M13 containing a kanamycin resistance gene and the P15A origin of replication that allows the genome to replicate as a plasmid in *E. coli* (48). A fully assembled phage particle contains five copies of the pIII protein, but since the wild type pIII gene from helper phage has superior expression levels compared to the phagemid-encoded pIII-antibody fusion gene, the majority of the produced phage population is expressed without a pIII-antibody fusion. However, only a portion of the population will contain monovalent display of the pIII-antibody fusion, with polyvalent display being much less frequent (49). The hyperphage system, which uses a helper phage lacking the pIII gene has been utilized for antibody pIII-antibody polyvalent display, because only the pIII-antibody gene of the phagemid is encoded (50). Nevertheless, monovalent display is the most popular display system because it allows for selection of higher affinity antibodies, avoiding the avidity effect of polyvalent display (43–46).

## Biopanning for Target-Specific Antibodies

**Figure 3:**
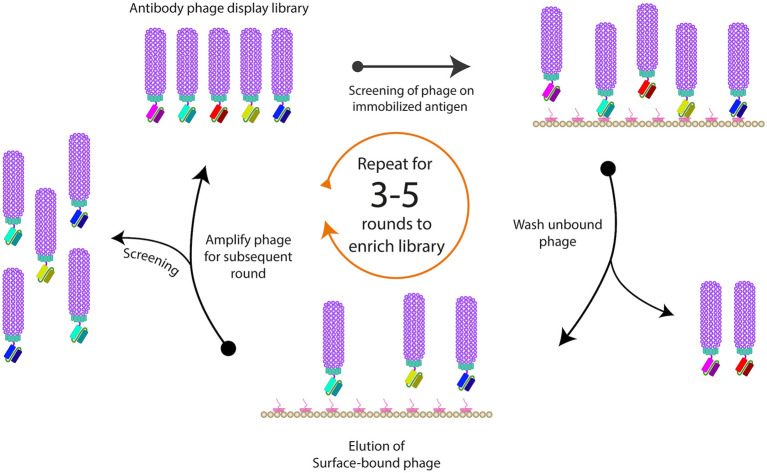
Schematic representation of phage biopanning. This is the basic method for sequential affinity screening of the phage display libraries for specific binding phage from a large excess of non-binding clones is often referred to as “panning or biopanning.” The phage antibody selection involves the immobilization of the ligand of interest on a solid support, followed by applying the phage display library (in the form of purified virions) to the immobilized ligand to allow binding of specific variants. To eliminate the adherent non-binders, multiple rounds of washing are usually performed, and remaining bound phages are eluted and re-amplified. At least three rounds of biopanning are usually required in order to amplify the binding variants and to exclude any non-specific binders.

When purified antigens are available, they can be presented to a phage antibody library by immobilization on solid surfaces, such as nitrocellulose membranes, polystyrene tubes or plates, magnetic beads or column matrices (51–53). The use of blocking agents, such bovine serum albumin (BSA), milk or casein can block the remaining sites present on the solid surface to prevent non-specific phage binding to the surface (54, 55). After the phage library is exposed to the immobilized antigens, unbound phages are usually washed away (Figure 3). Such washing step is critical to remove non-specific binders, and to allow for some control over binding properties by manipulating the wash buffer and stringency of washing. For example, long wash times can be incorporated to ensure only clones with slow dissociation rates are selected. Detergents are usually included in wash buffers, but they can also be altered for factors, such as pH and salt concentration. The washing steps are gradually increased with every round of biopanning to increase the stringency in order to isolate higher affinity phage clones (46, 56).

To recover high-affinity phage antibodies from immobilized antigens, different elution conditions, including change in pH, proteolytic cleavage or competition with free antigens have been used. For pH elution, either acidic buffers, such as glycine or citric acid (52, 57), or alkaline triethylamine (TEA) can be used (51). It is crucial to neutralize the pH of eluted phage antibodies to be around 8, to avoid degradation of the phage and maintain infectivity. Some libraries have a cleavage site introduced between the antibody and the pIII protein to facilitate elution by using proteases, such as Genenase I or trypsin (58, 59).

After several rounds of biopanning, the pool of phages isolated from each round is tested, usually by ELISA, to determine if there is an enrichment of phage binders toward the specific antigen within the polyclonal pool. The polyclonal ELISA involves immobilizing antigen onto microtitre plates, followed by addition of various dilutions of the phage pool from each round and then detection of bound phage using an anti-M13 phage antibody. Individual clones from the round of biopanning exhibiting the maximum enrichment level are then further screened by ELISA to determine individual phage isolates with high specificity toward the antigen of interest. The procedure involves growing single colonies cell glycerol stock from the last performed biopanning round in a 96-well plate format, before adding the helper phage to induce production of phage particles. The positive clones derived from this experiment can then be analyzed by restriction fragment length polymorphism to determine the number of unique clones, or by sequencing which also determines the CDRs for both heavy and light chains (60). Once positive clones are isolated, downstream applications would determine how they are further processed. For example, a scFv gene from a phage clone can be re-cloned into a bacterial expression vector for large scale production or reformatted into a full mAb by inserting the variable regions into expression vectors containing the antibody constant regions (61). *In vitro* affinity maturation using mutated libraries of lead phage clones can also be conducted in order to increase the affinity and the stability of the selected antibodies (62, 63).

Although biopanning of immobilized antigen on solid surfaces is robust, it is often limited by the availability of purified protein and the possibility of its altered conformation when attached to solid surfaces. Therefore, alternative methods, such as in-solution biopanning, followed by affinity capture of antigens tagged with biotin (64), or calmodulin binding peptide have been used (65).

Some membrane proteins (66) are poorly soluble in an aqueous media, and due to their complexity, they do not form properly during recombinant expression (67). They might form aggregates and lose their tertiary structures when coated on immunotubes before biopanning (68–70), which as a result might lead to generate antibody binders that recognize epitopes that are not naturally exposed (71, 72). Thus, cell-based biopanning is often utilized to maintain membrane proteins native conformation (73–76). It can be applied to retrieve antibodies that are specific for either known or unknown antigens on cells surface, and it can be performed in case of unavailability of the targeted antigen in pure form (77–80). Furthermore, cell-based biopanning strategies allow for selecting binders to a specific conformational state of a cell surface receptor (81–85). Cell-based microselection approach, can be applied to retrieve unique binders, and identify novel biomarkers that are exclusively expressed on rare cells within a heterogeneous solution (86, 87).

The latest advancements in next-generation sequencing (NGS) technologies, bioinformatics and nanotechnology have tremendously improved the high-throughput screening of antibody discovery (88–91). For instance, a report from Raftery et al. (92) described a rapid selection of scFv-phage (PhageXpress) using electrohydrodynamic-manipulation of a solution containing phage library particles in combined with Oxford Nanopore Technologies' MinION sequencer. After a single round of biopanning and within 2 days compared to several weeks if applying traditional biopanning, they were able to identify 14 anti-dengue virus non-structural protein 1 scFv. Adopting similar approaches will significantly reduce the time and amount of laborious lab work required to discover putative antibodies, which are major obstacles in the traditional biopanning method, and will help accelerate developing therapeutic monoclonal antibodies during emerging infectious outbreaks (93).

## Phage Display Libraries as an Antibody Discovery Platform

Antibody phage display is a versatile, *in vitro* selection technology that can be utilized to discover high affinity antibodies specific to a wide variety of antigens (94). However, specificity and high affinity are not the only attributes that account for successful therapeutic antibodies. Other antibody quality attributes, such as solubility, viscosity, expression yield, and thermal and long-term stability are vital to ensure the success of mAb lead candidates in biomanufacturing and clinical trials (95, 96). These biophysical properties of antibodies are strongly dependent on their amino acid sequences (97). Some mAbs might have poor developability profiles because of high immunogenicity, physicochemical instability, self-association, high viscosity, poly-specificity, short half-life, and poor expression (98, 99). For instance, low solubility can lead to issues during biomanufacturing (100–102), and could affect mAb potency, bioavailability and immunogenicity (103, 104). High thermal stability is crucial to maintain structural and functional integrity, and intrinsic properties, under different temperatures (105, 106). Furthermore, aggregation is one of the main challenges that limit the advancement of therapeutic mAb due to immunogenicity concerns (107–110).

Despite the several advantages of antibody phage display, such as bypassing animal immunization, the ability to isolate antibodies against toxic or non-immunogenic antigens and the ability to generate conformation-specific antibodies, the vast majority of the approved therapeutic antibodies are derived from immunized mice technologies. This is because the filtration process that imposed by the immune system enables mammalians derived antibodies to have better biophysical attributes compared to antibodies generated by phage display (111). In agreement with this, Jain et al. has comprehensively analyzed the biophysical attributes for 46 FDA approved therapeutic antibodies and 89 in advance clinical trials (96). They found that antibodies directly discovered by phage display or engineered at some point by phage biopanning exhibit significant developability risks' properties compared to than those derived from immunized mice. Further investigations found that phage display derived therapeutic antibodies have higher self-interaction and poly-reactivity due to the higher percentage of aliphatic residues in their CDRs compared to the non-phage derived antibodies (112).

Additionally, antibodies selected form phage display libraries are not glycosylated, because they are produced in *E. coli*, as a result, some candidates when glycosylation occur during mammalian cells expression; their binding, biodistribution, or pharmacokinetics might be negatively impacted (113–116). Therefore, using eukaryotic display platforms like yeast and mammalian display would be beneficial. For instance, in addition to their ability to produce glycosylated proteins, yeast and mammalian antibody libraries can be constructed to display full-length antibodies as well as antibody fragments, such as scFvs and fragment antigen-binding region (Fabs) (117–126), allowing the isolation of high affinity antibodies with definitive biological characteristics (122, 123). For example, Parthiban et al. has developed mammalian libraries that display around 10 million clones in IgG-format on the surface of HEK293 cells using CRISPR/Cas9 or transcription activator-like effector nucleases (TALENs) (127). These libraries can act as a quality filter for different antibody developability aspects, and to provide a very early insight into developability problems, such as aggregation and cross-reactivity. Each display system has its advantages and disadvantages, however, determining those are beyond the intended scope of this review, which is about the most commonly used type of antibody display, the phage display. Therefore, it is vital to generate phage libraries that allow for the isolation of highly specific and diverse mAbs with high affinity against diverse antigens with optimal developability potential (128–130).

Currently, as a common practice in industrial pipelines, biopharmaceutical companies are implementing extensive developability assessments to determine the biochemical and biophysical features of antibody candidates to help identifying candidates with more favorable biophysical properties and to avoid difficulties during the downstream process (131, 132). For example, *in silico* platforms, such as the Therapeutic Antibody Profiler (TAP) tool are used as a flagging system to predict mAbs with poor developability profiles by identifying anomalous values compared with therapeutic mAbs in clinical-stages. Indeed, features within the variable regions of mAbs, such as the total CDRs length, high hydrophobicity of V_H_ and V_L_ chains, lack of net charge symmetry, and/or the presence of patches of positive and negative charges were computationally predicted to be key factors in developability profiles of mAbs (133).

The probability of isolating high affinity, and more diverse mAbs that specifically bind random epitopes, increases significantly when biopanning campaigns are performed using larger antibody libraries. Library diversity is judged by how many functional antibody fragments are able to identify as many different antigens as possible (134). The bacterial transformation step during library construction; however, is a main practical bottleneck that limits the size of the library from exceeding 10^11^ antibody variants, even after optimization and performing numerous electroporation steps.

Ideally, antibody phage display libraries should not only be large and diverse, but also should display antibody variants as functional fragments. Issues related to the nucleotide sequences, such as the presence of stop codons, or the addition/deletion of nucleotides can occur during the library construction (135–137). These issues might inhibit the production of functional pIII-antibody fusions or change the reading frame of the antibody gene sequence, which could negatively affect their biophysical characteristics. Some in-frame antibody genes might also have poor expression levels from their phagemid, or produce aggregated, misfolded, or toxic antibody fragment to *E. coli* (138–141). However, such variants are usually displayed in lower percentage compared to other variants or phages that do not display any fusion protein.

## Types of Antibody Phage Display Libraries

Phage libraries generated from human rearranged V-gene repertoires are constructed from mRNA or RNA extracted from B cells of immunized or naïve donors (Figure 2) (73, 142–144). Construction of immunized or naïve libraries involves using reverse transcription polymerase chain reaction (RT-PCR) to prepare the cDNA template. This is followed by the amplification of the repertoire of V_L_ and V_H_ genes by PCR, before cloning into the phagemid.

Immunized libraries are constructed from lymphoid tissues of individuals who carry a particular disease, such as metastatic cancer or particular infection, or have been immunized with a particular antigen (145–149). Such libraries are characterized by a biased antibody repertoire toward specific targets. Additionally, those antibodies tend to have much higher affinities for the desired antigen than antibodies isolated from naïve libraries of comparable size, because the V_H_ and V_L_ gene fragments have undergone the natural *in vivo* affinity maturation process (150). Naïve libraries, on the other hand, represent the germline diversity of antibody repertoire. These libraries are generated from healthy donor's mRNA or RNA without bias toward a particular disease state, and are used to yield mAbs against unlimited range of antigens (151). To generate a highly diverse naïve antibody phage library, it is recommended to use a large pool of donors from diverse ethnic groups, and to maximize the efficiency of antibody gene amplification in the process of library construction (152–155).

The CDRs play a significant role in antigen recognition (156), although some of the non-CDRs residues contribute to the antibody-antigen interaction (157). Each CDR loop contributes differently to antibody-antigen binding, and each residue within each CDR loop plays a different role in this interaction (158, 159). Among all the six CDR loops, the V_H_ CDRs, especially V_H_'s CDR3 (CDRH3), are more frequently involved in the antigen binding than those in the light chain (160, 161). The CDRH3 loop, which exists in a variety of different lengths (5–30 amino acids), is of particular importance due to its substantial impact on the canonical conformation and antigen binding compared to the other CDRs (156, 162–165). Noteworthy, the loop length of CDRH3 does not only affect the specificity and affinity of the antibody for its specific antigen, but also affects the nature of the binding of other CDRs. Specifically, for antibodies with long CDRH3 loops, these loops are responsible for most of the antibody-antigen interactions, while in antibodies with short CDRH3 loops, other CDRs loops usually assist in antigen binding (156). Thus, CDRH3 plays a major role in recognizing diverse targets, and generating interactions with acceptable affinity (166, 167).

The diversity of the V-gene segments can be designed and synthesized artificially by CDRs randomization. These libraries can be fully synthetic or semisynthetic. Synthetic libraries are made to maximize antibodies' functionality by making a large and highly diverse phage repertoire. This is usually achieved *in vitro* by using PCR and oligonucleotides to create a random integration of the CDRs as well as introduction of different CDRH3 loop sequences and lengths without disrupting the folding of the V regions (94, 168). Semisynthetic libraries combine natural and synthetic antibody diversity. They are constructed from non-rearranged V-genes from pre-B cells, or an antibody framework with randomization of the CDRH3 or several CDRs utilizing degenerated oligonucleotides (128, 169, 170).

## Phage Display-Derived mAbs

Data collected for this review were obtained from different sources including PubMed, the clinical trial database (www.clinicaltrials.gov), patents, company websites, and international ImMunoGeneTics information system (www.imgt.org). A selection of phage display-derived therapeutics was described in great detail previously (171, 172), yet we here present an updated and comprehensive review of phage display-derived mAbs.

**Figure 4:**
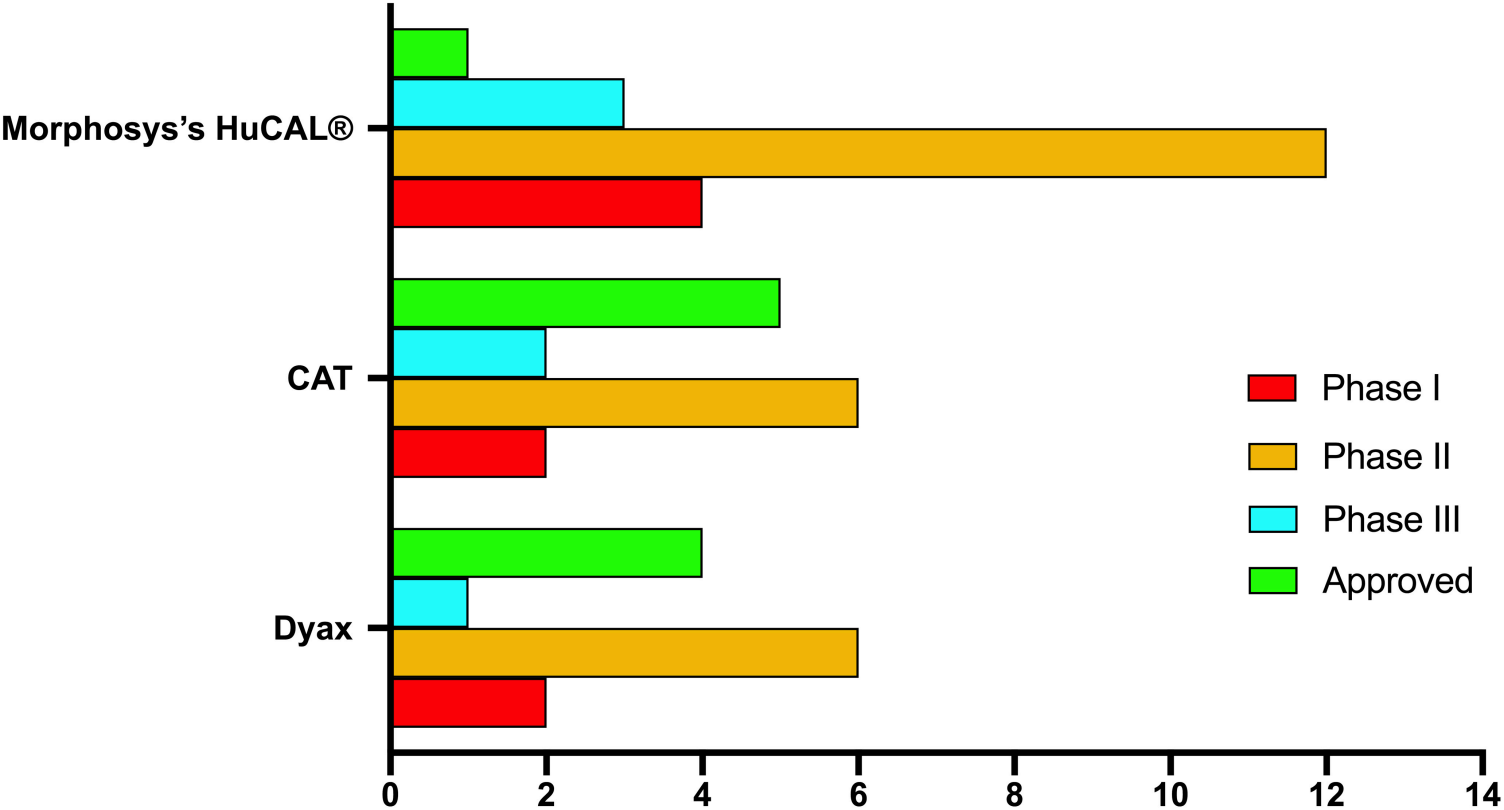
Highest development phase achieved for antibodies isolated from various major company-owned libraries.

Two decades after McCafferty and Winter's seminal report in 1990, more than 70 phage–derived mAbs entered clinical studies, and 14 of them have been approved. The majority of these antibodies are generated by three company-owned libraries, Cambridge Antibody Technology (CAT), Dyax and MorphoSys's human combinatorial antibody libraries (HuCAL®) (Table 1, Figure 4). MorphoSys's HuCAL® has the highest number of mAbs (20 mAbs), wherein 19 are under clinical development, and one (Tremfya™) is approved. The majority of the MorphoSys's HuCAL® derived mAbs (12 mAbs) are in phase II clinical trials. CAT (AstraZenica) has the second highest number of phage derived mAbs (15 mAbs) in clinical trials, and the highest number of approved mAbs including Humira®, Benlysta®, Lumoxiti™, ABthrax®, and Gamifant®. Dyax has 13 mAbs in which four of them have been approved; Bavencio®, Portrazza™, Cyramza®, Takhzyro®.

**Table 1:** The design, construction, and features of some major company-owned antibody-phage libraries.

**Library**	**Type**	**Source**	**Size**	**Note**	**References**
CAT-BMV	Naïve scFv	PBL, tonsils	1.4 × 10^10^	A total of 43 healthy donors	(173)
CAT-CS	Naïve scFv	Spleen, fetal liver	1.29 × 10^11^	cDNA from 160 donors	(174)
CAT-BMV	Naïve scFv	Spleen	1.2 × 10^11^	Derived from the germline V_H_ gene DP47 from CAT-BMV library, and B-cell-derived V_L_ and CDRH3 from CAT-CS library	(175)
Dyax	Naïve Fab	PBL, spleen	3.7 × 10^10^	PBL from 4 healthy donors, and part of a tumor-free spleen removed from a patient with gastric carcinoma	(176)
Morphosys's HuCAL®	Synthetic Fab	—	2.1 × 10^09^	CDRH3 and CDRL3 were diversified by TRIM	(177)
Morphosys's HuCAL GOLD®	Synthetic Fab	—	1.6 × 10^10^	All CDRs were diversified by TRIM	(178)
Morphosys's HuCAL PLATINUM®	Synthetic Fab	—	4.5 × 10^10^	HuCAL PLATINUM® is an advanced version of HuCAL GOLD®. All CDRs were diversified by TRIM, with additional sequence optimization to enhance mammalian cells expression and avoid undesirable motifs	(179)

*CAT, Cambridge Antibody Technology human antibody phage display library; HuCAL, Human combinatorial antibody library; PBL, Bone marrow, peripheral blood lymphocytes; TRIM, trinucleotide-directed mutagenesis method*.

**Figure 5:**
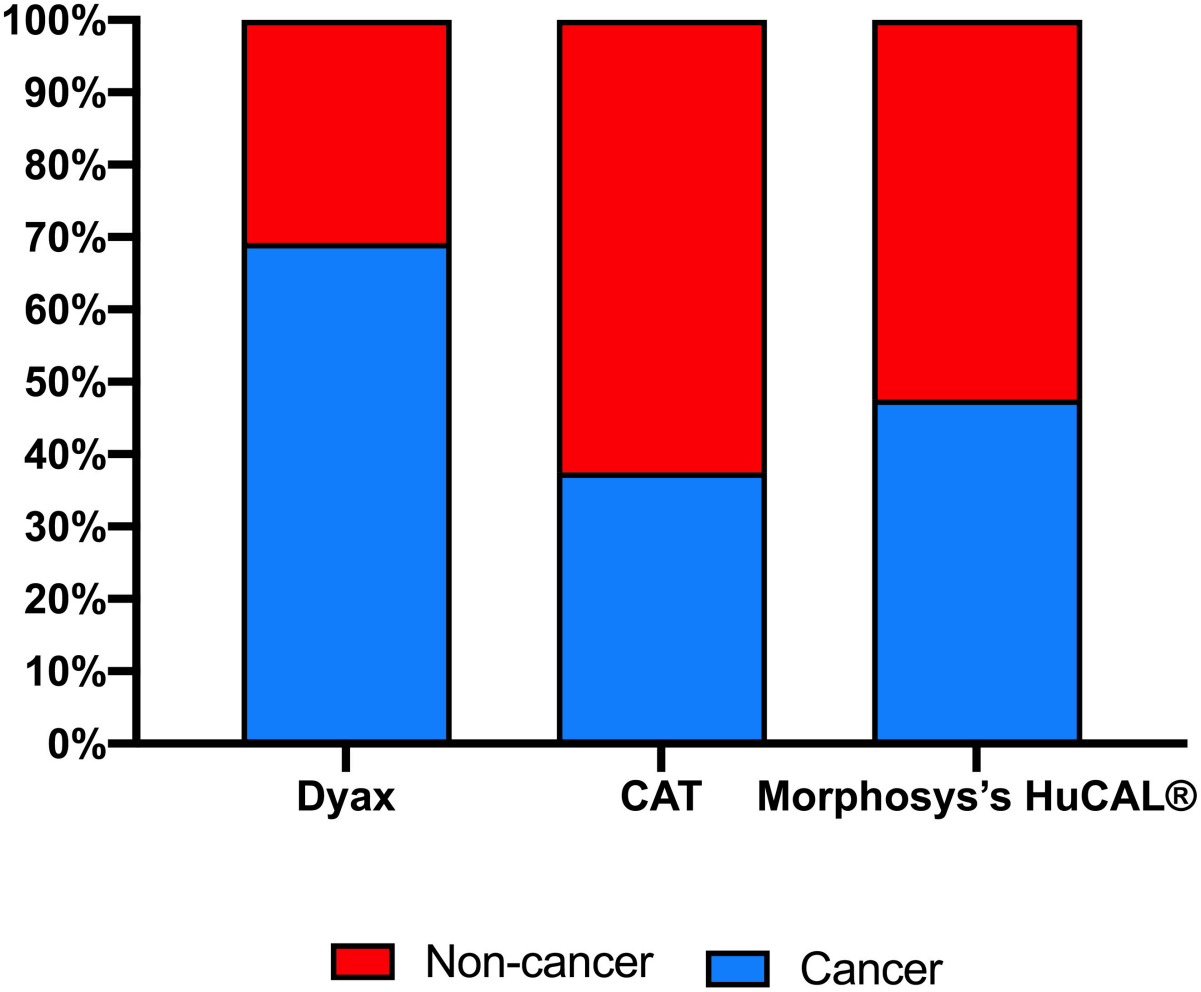
Indications for antibodies isolated from various major company-owned libraries. Clinical domains (cancer vs. non-cancer) of the approved or in clinical trials antibody-derived phage display.

Therapeutic mAbs from phage libraries can be successfully isolated to treat cancer, and non-cancer medical conditions, such as inflammatory, optical, infectious, or immunological diseases (Table 2). However, some of the aforementioned major libraries have a favorable therapeutic area of application. More mAbs for non-cancer indications in comparison to cancer indications (~63 vs. ~37%) were developed using CAT libraries. Among all the five approved CAT derived mAbs, Lumoxiti™ is the only one that is indicated to treat cancer. Unlike CAT, Dyax libraries have been remarkably useful in the development of therapeutic mAbs for oncology at the expense of non-oncology indications (~70 vs. ~30%), in which three out of the four approved mAbs are anti-cancer agents (Figure 5). MorphoSys's HuCAL® libraries have almost equally contributed to both cancer and non-cancer indications (Figure 5).

**Table 2:** A list of phage display-derived therapeutic antibodies that either are approved or have been investigated clinical trials.

**Product name/Brand name**	**Antibody format**	**Target antigen**	**Antibody phage display type**	**Phage display technology**	**Clinical domain**	**Indication(s)**	**Highest development phase**	**Sponsor company**
Adalimumab (D2E7)/Humira®	IgG1-κ	TNFAα	Humanization by Phage display guided selection using a naïve scFv-phage library (180)	CAT (51, 173–175)	Immunology, and inflammation (181–184)	RA	Approved 2002	AbbVie
						PSA	Approved 2005	
						AS	Approved 2006	
						CD	Approved 2007	
						Psoriasis, severe chronic plaque	Approved 2008	
						JIA	Approved 2008	
						UC	Approved 2012	
						HS	Approved 2015	
						Fingernail psoriasis	Approved 2017	
					Ophthalmology (185)	Uveitis	Approved 2016	
Adecatumumab (MT201)	IgG1	EpCAM	Guided selection of light chain, naïve (IgD), Fab (186)	Micromet AG	Oncology (187–190)	Breast cancer, prostate cancer, colorectal cancer	Phase II	Amgen
1D09C3	IgG4	HLA-DR	Synthetic scFv (191)	Morphosys's HuCAL® (177)	Oncology (192, 193)	HL, myeloma	Phase I	GPC Biotech AG
Anetumab ravtansine (unconjugated BAY 86-1903, conjugate BAY 94-9343)	IgG1-λ conjugated to the maytansinoid tubulin inhibitor DM4	MSLN	Synthetic Fab (194)	Morphosys's HuCAL GOLD® (178)	Oncology (195, 196)	Mesothelioma, mesothelin-expressing ovarian cancer, non-small cell lung cancer and pancreatic cancer	Phase II	Bayer
Amatuximab (MORAb-009)	IgG1-κ	MSLN	Immune scFv (197, 198)	NCI, US	Oncology (199, 200)	Mesothelioma, mesothelin-expressing pancreatic cancer	Phase II	Eisai Inc
Atezolizumab (MPDL3280A)/Tecentriq™	IgG1-κ	PD-L1	Antibody phage display library (201, 202)	Genentech	Oncology (203–208)	Renal cancer	Phase I	Roche
						Solid tumors	Phase II	
						SCLC	Phase III	
						Malignant melanoma	Phase III	
						Mesothelioma (PMID: 32206576)	Phase III	
						Bladder cancer	Phase III	
						RCC	Phase III	
						HCC	Phase III	
						NSCLC	Approved 2016	
						Urothelial Carcinoma	Approved 2016	
						Urothelial bladder cancer	Approved 2017	
						Breast cancer	Approved 2019	
Avelumab/Bavencio®	IgG1-λ	PD-L1	Naïve Fab (209)	Dyax (176)	Oncology (210–216)	Ovarian cancer	Phase III	Merck Serono/Pfizer
						Gastric cancer	Phase III	
						NSCLC	Phase III	
						Solid tumors	Phase I	
						mMCC, metastatic urothelial carcinoma	Approved 2017	
						RCC	Approved 2019	
Belimumab (LymphoStat-B)/Benlysta®	IgG1-λ	BLyS	Naïve scFv (217)	CAT	Immunology, and inflammation (218–220)	SLE	Approved 2011	GSK/HGSI
						Vasculitis	Phase III	
Bertilimumab (CAT-213)	IgG4-κ	CCL11, eotaxin-1	Naïve scFv (221)	CAT	Immunology (222–225)	Severe ocular allergies	Phase I	Immune Pharmaceuticals
						CD	Phase II	
						UC	Phase II	
						Bullous pemphigoid	Phase II	
Bimagrumab (BYM338)	IgG1-λ	ActRII	Synthetic human Fab (226)	Morphosys's HuCAL GOLD®	Endocrinology, and Immunology (227)	Type 2 diabetes	Phase II	Novartis
					Immunology, and myology (228–231)	Cachexia	Phase II	
						Sporadic inclusion body myositis	Phase III	
						Musculoskeletal diseases	Phase II	
						Sarcopenia	Phase II	
Carlumab (CNTO 888)	IgG1-κ	CCL2/MCP-1	Synthetic Fab (232)	Morphosys's HuCAL GOLD®	Oncology (233, 234)	Solid tumors	Phase I	Janssen
					Oncology (235)	Prostate cancer	Phase II	
					Pulmonary, and Respiratory diseases (236)	Pulmonary fibrosis	Phase II	
Cixutumumab (IMC-A12)	IgG1-λ	IGF1R	Naïve Fab (237)	Dyax	Oncology (238–240)	NSCLC, HCC, solid tumors	Phase II	Eli Lilly/ImClone
Foravirumab (CR4098)	IgG1-κ	Rabies virus glycoprotein	Immune scFv (241, 242)	Crucell	Immunology, and Infectiology (5)	Prophylaxis of rabies	Phase III	Sanofi
Fresolimumab (GC-1008)	IgG4-κ	TGFβ	Naïve scFv (243)	CAT	Oncology, and immunology (244–247)	Scleroderma, metastatic breast cancer, NSCLC, fibrosis, focal segmental glomerulosclerosis	Phase II	Genzyme/Sanofi
Ixekizumab (LY2439821)/Taltz®	IgG4-κ	IL17A	Immune Fab (248)	Eli Lilly	Immunology (249–252)	RA	Phase II	Eli Lilly
						Psoriasis	Approved 2016	
						PSA	Approved 2017	
						AS	Approved 2019	
Mapatumumab (HGS-ETR1)	IgG1-λ	TRAIL-1	Naïve scFv (253)	CAT	Oncology (254–257)	Multiple myeloma, colorectal cancer, NSCLC, NHL, cervical cancer	Phase II	GSK/HGSI
Mavrilimumab (CAM-3001)	IgG4-λ2	GM-CSFRα	Naïve scFv (258)	CAT	Immunology (259–261)	RA, GCA, COVID-19	Phase II	MedImmune /AstraZeneca
Moxetumomab pasudotox (CAT-8015)/Lumoxiti™	Murine IgG1 dsFv and a *Pseudomonas* exotoxin A	CD22	Affinity maturation of BL22 by phage display (122, 262)	CAT	Oncology (263, 264)	HCL	Approved 2018	MedImmune /AstraZeneca
Namilumab (MT203)	IgG1-κ	GM-CSF	Humanization rat scFv by Phage display guided selection using a naïve human scFV-phage library (human V_H_ contains rat CDR3) (265, 266)	Micromet AG/CAT	Immunology (267–269)	RA, AS, psoriasis	Phase II	Takeda
Necitumumab (IMC-11F8)/Portrazza™	IgG1-κ	EGFR	Naïve Fab (270, 271)	Dyax	Oncology (272–275)	NSCLC	Approved 2015	Eli Lilly/AstraZeneca
						Colorectal cancer	Phase II	
						Solid tumors	Phase I	
Opicinumab (BIIB-033, Li81)	IgG1-κ	LINGO 1	Naïve Fab (276)	Dyax	Immunology, and inflammation (277, 278)	MS, and Optic neuritis	Phase II	Biogen
Tanibirumab (Olinvacimab, TTAC-0001)	IgG1-κ-λ	VEGFR2	Naïve scFv (279, 280)	PharmAbcine	Oncology (281, 282)	Solid tumors	Phase I	PharmAbcine
						Glioblastoma	Phase II	
Utomilumab (PF-05082566)	IgG2-λ	4-1BB (CD137)	Synthetic Fab (283)	Morphosys's HuCAL GOLD®	Oncology (284–288)	Solid tumors	Phase I	Pfizer
						Breast cancer, B-cell lymphoma, NHL	Phase II	
Ganitumab (AMG 479)	IgG1-κ	IGF-1R	Naïve Fab (289, 290)	Dyax	Oncology (291–293)	Metastatic colorectal cancer	Phase II	Amgen
						Pancreatic cancer, metastatic Ewing Sarcoma	Phase III	
AMG 780	IgG2	Ang-1 and−2	Naïve Fab (294)	Dyax	Oncology (295)	Solid tumors	Phase I	Amgen
Caplacizumab (ALX-0081)/Cablivi™	Humanized V_H_-V_H_. Genetically linked by a triple-alanine linker	VWF A1 domain	Immune, camelidae-derived nanobody library (296–298)	Nanobody®	Cardiology, and hematology (299, 300)	aTTP	Approve 2018	Sanofi/Ablynx
Ramucirumab (IMC1121B)/Cyramza®	IgG1-κ	VEGFR2	Naïve Fab (301, 302)	Dyax	Oncology (303–310)	Tumors vasculature	Phase I	Eli Lilly
						Solid tumors	Phase II	
						Breast cancer, Bladder cancer	Phase III	
						Gastric cancer, NSCLC	Approved 2014	
						Colorectal cancer	Approved 2015	
						HCC	Approved 2019	
Ranibizumab (Fab-12 variant Y0317)/Lucentis®	Fab-IgG1-κ	VEGFA	Affinity maturation of bevacizumab (311, 312) by phage display (313)	Genentech	Immunology, and Ophthalmology (314–319)	nAMD	Approved 2006	Roche/Novartis
						MEfRVO	Approved 2010	
						DME	Approved 2012	
						CNV	Approved 2016	
						Diabetic retinopathy	Approved 2017	
MOR202	IgG1-λ	CD38	Synthetic human Fab (320)	Morphosys's HuCAL GOLD®	Oncology (321)	MM	Phase II	Morphosys/I-MAB Biopharma
Darleukin (bifikafusp alfa, L19-IL2)	L19 scFv-IL2 fusion, diabody	EDB-FN	Semi-synthetic scFv (322)	Alessandro Pini's library (168, 322)	Oncology (323, 324)	Solid cancers	Phase I/II	Philogen
						Metastatic melanoma	Phase III	
Fibromun (Onfekafusp alfa, L19-TNF)	L19 scFv-TNFα fusion, diabody	EDB-FN	Semi-synthetic scFv (325)	Alessandro Pini‘s library	Oncology (324, 326, 327)	Glioma	Phase II	Philogen
						Metastatic Melanoma, Soft tissue sarcoma, glioma	Phase III	
Radretumab (^131^I-labeled L19SIP)	[L19 scFv-IgE-CH4-Iodine-131 fusion]2	EDB-FN	Semi-synthetic scFv (328)	Alessandro Pini's library	Oncology (329, 330)	Solid tumors, lymphomas	Phase II	Philogen
Raxibacumab/ABthrax®	IgG1-λ	Anthrax PA, *Bacillus anthracis*	Naïve scFv (331)	CAT	Infectious diseases (332)	Inhalation anthrax	Approved 2012	GSK/HGSI
Otilimab (MOR04357, GSK3196165)	IgG1-λ	GM-CSF	Synthetic Fab (333)	Morphosys's HuCAL GOLD®	Immunology (260, 334, 335)	OS	Phase II	GSK
						RA	Phase III	
Seribantumab (MM-121)	IgG2-λ	HER3	Naïve Fab (336, 337)	Dyax	Oncology (248, 338, 339)	Ovarian cancer, breast cancer, NSCLC	Phase II	Sanofi/Merrimack
Tralokinumab (CAT-354, BAK 1.1)	IgG4-λ	IL13	Naïve scFv (340, 341)	CAT	Immunology (342, 343)	Asthma, atopic dermatitis	Phase III	MedImmune /AstraZeneca/LEO pharma
Ianalumab (VAY736, B-1239)	Defucosylated IgG1-κ	BAFF-R	Synthetic Fab (344, 345)	Morphosys's HuCAL GOLD®/ POTELLIGENT® technology	Immunology (346–348)	CLL	Phase I	Novartis
						pSS, MS	Phase III	
Teleukin (F16-IL2)	F16 scFv-IL2 fusion, diabody	A1 domain of tenascin-C	Synthetic scFv (349, 350)	ETH-2 library (351)	Oncology (352–354)	AML	Phase I	Philogen
						MCC, breast cancer	Phase II	
Xentuzumab (BI 836845)	IgG1-λ	IGF-I, IGF-II	Synthetic Fab (355)	Morphosys's HuCAL GOLD®	Oncology (356–359)	NSCLC, solid tumors	Phase I	Boehringer Ingelheim
						Breast cancer	Phase II	
Setrusumab (BPS-804, MOR05813)	IgG2-λ	SOST	Synthetic Fab (178, 360)	Morphosys's HuCAL GOLD®	Supportive therapy (361–363)	OI, HPP, post-menopausal women with low BMD	Phase II	Mereo BioPharma/Novartis
IMC-3C5 (hF4–3C5, LY3022856)	IgG1	VEGFR-3	Naïve Fab (364)	Dyax	Oncology (365)	Solid tumors	Phase I	Eli Lilly/ImClone
Aprutumab (BAY 1179470)	IgG1-λ	FGFR2	Semisynthetic scFv (366)	BioInvent's n-CoDeR™ library (367)	Oncology (368)	Solid tumors	Phase I	Bayer HealthCare
BAY 1093884	IgG2	TFPI	Synthetic Fab (369–371)	Morphosys's HuCAL GOLD®	Hematology (372)	Hemophilia A and B	Phase II	Bayer HealthCare
BAY 1213790	IgG1	FXI	Naïve Fab (373, 374)	Dyax	Hematology (375)	VTE	Phase II	Bayer HealthCare/XOMA
CNTO-6785	IgG1-λ	IL17A	Synthetic Fab (376)	Morphosys's HuCAL GOLD®	Immunology (377, 378)	COPD, RA	Phase II	Janssen
CNTO-3157	IgG4-κ	TLR-3	Synthetic Fab (379)	Morphosys's HuCAL GOLD®	Immunology (380)	Asthma	Phase I	Janssen
Briakinumab (ABT-874)	IgG1-λ	IL12 and IL23	Naïve scFv (381, 382)	CAT	Immunology (383, 384)	MS	Phase II	Abbott
						Psoriasis	Phase III	
BHQ880	IgG1-λ	DKK1	Synthetic Fab (385)	Morphosys's HuCAL GOLD®	Oncology (386, 387)	MM	Phase II	Novartis
BI-1206 (6G11)	IgG1	FcγRIIB (CD32B)	Semisynthetic scFv (388)	BioInvent's n-CoDeR™ library	Oncology (389)	NHL, CLL	Phase I/II	BioInvent
Dekavil (F8-IL10)	F8 scFv-Interleukin 2 (IL2) fusion, diabody	EDA-FN	Synthetic scFv (390)	ETH-2 library	Immunology (391)	RA	Phase II	Philogen/Pfizer
Gancotamab (MM-302, C6.5)	scFv-λ targeted liposomal doxorubicin	HER2	Naïve scFv (392, 393)	CAT	Oncology (394, 395)	Breast cancer	Phase II	Merrimack
Guselkumab (CNTO1959)/Tremfya™	IgG1-λ	IL23, subunit P19	Synthetic Fab (396, 397)	Morphosys's HuCAL GOLD®	Immunology (398–400)	RA, palmoplantar pustulosis	Phase III	Janssen
						Psoriasis	Approved 2017	
Lupartumab amadotin (BAY 1129980)	Antibody-drug conjugate (ADC), IgG1-λ conjugated with auristatin W derivative	LYPD3 (C4.4A)	Semisynthetic scFv (401)	BioInvent's n-CoDeR™ library	Oncology (401)	Solid tumors	Phase I	Bayer
Lanadelumab (DX-2930)/Takhzyro®	IgG1-κ	pKal	Naïve Fab (402)	Dyax	Immunology (403, 404)	HAE	Approved 2018	
Lexatumumab (HGS-ETR2)	IgG1-λ	TRAIL-R2 (DR5)	Naïve scFv (79, 253)	CAT	Oncology (405, 406)	Solid tumors	Phase I	HGS
Oleclumab (MEDI9447)	IgG1-λ	CD73	Naïve scFv (407)	CAT	Oncology (408–410)	Solid tumors	Phase I	AstraZeneca /MedImmune
						NSCLC, breast cancer	Phase II	
Tarextumab (OMP-59R5)	IgG2-κ	Notch2/3	Synthetic Fab (411)	Morphosys's HuCAL GOLD®	Oncology (412–415)	Solid tumors	Phase I	GSK/OncoMed
						Pancreatic Cancer, SCLC	Phase II	
Elgemtumab (LJM716)	IgG1-mk	HER3	Synthetic Fab (416, 417)	Morphosys's HuCAL GOLD®	Oncology (418–421)	Breast cancer, gastric cancer	Phase I	Novartis
						Esophageal cancer, HNSCC	Phase I/II	
Gantenerumab (R1450)	IgG1-κ	Amyloid-β (Aβ)	Synthetic Fab (422)	Morphosys's HuCAL®-Fab1 library (170)	Neurology (423)	Alzheimer's disease	Phase III	Roche
Vantictumab (OMP-18R5)	IgG2-λ	FZD family receptor, including FZD1, FZD2, FZD5, FZD7, and FZD8	Synthetic Fab (424)	Morphosys's HuCAL GOLD®	Oncology (425, 426)	Breast cancer; NSCLC, pancreatic cancer	Phase I	OncoMed/Bayer
MEDI4212	IgG1-λ	IgE	Naïve scFv (427)	CAT	Immunology (428)	Asthma	Phase I	MedImmune/ AstraZeneca
Drozitumab (Apomab, PRO95780)	IgG1-λ	TRAIL-R2 (DR5)	scFv (429)	Genentech	Oncology (430–432)	Solid tumors	Phase I	Genentech
						NHL, NSCLC	Phase II	
Tesidolumab (LFG316)	IgG1-λ	Complement 5 (C5)	Synthetic Fab (433)	Morphosys's HuCAL GOLD®	Inflammation, and Ophthalmology (434, 435)	Geographic Atrophy, uveitis, panuveitis, PNH, AMD	Phase II	Novartis
Emapalumab (NI-0501)/Gamifant®	IgG1-λ	Interferon-gamma	Naïve scFv (436)	CAT	Hematology (437)	HLH	Approved 2018	NovImmune SA
Imalumab (Anti-MIF, BAX69)	IgG1-κ	MIF	Naïve Fab (438)	Dyax	Oncology (439, 440)	Colorectal cancer, malignant ascites, ovarian cancer	Phase II	Baxter/Takeda
Bersanlimab (BI-505)	IgG1-λ	ICAM1	Semisynthetic scFv (441)	BioInvent's n-CoDeR™ library	Oncology (442)	MM	Phase II	BioInvent
Orticumab (BI-204/MLDL1278A)	IgG1-λ	oxLDL	Semisynthetic scFv (443)	BioInvent's n-CoDeR™ library	Cardiology (441)	Atherosclerosis	Phase II	BioInvent/ Genentech
PC-mAb (M99-B05)	IgG1-κ	ChoP	Naïve human Fab (444, 445)	Dyax	Cardiology (446)	Myocardial infarction	Phase II	Athera Biotechnologies/ Dyax
m102.4	IgG1-κ	Ephrin-B2 and -B3 receptor binding domain of the henipavirus G envelope glycoprotein	Affinity maturation of clone m102 by phage display (447, 448)	NCI	Infectious diseases (449)	NiV and HeV infections	Phase I	Profectus Biosciences
Cusatuzumab (ARGX-110)	Defucosylated IgG1-λ	CD70	Immunized lama Fab-based library followed by antibody humanization using synthetic libraries with phage expressing germlined Fabs (450–452)	SIMPLE Antibody™/ POTELLIGENT® technology	Oncology (453, 454)	AML	Phase II	Argenx/Janssen
						Solid tumors and hematologic malignancies	Phase I	
ARGX-111	Defucosylated IgG1-λ	c-MET	Immunized lama Fab-based libraries followed by antibody humanization by re-assembling into human IgG1 and λ light chain constant domains (455–457)	SIMPLE Antibody™/ POTELLIGENT® technology/NHance™ technology	Oncology (458)	Solid tumors	Phase I	Argenx

*Data current as of May 20, 2020*.

*TNFAα, Tumor necrosis factor-alpha; RA, Rheumatoid arthritis; PSA, Psoriatic arthritis; AS, Ankylosing spondylitis; CD, Crohn's disease; JIA, Juvenile Idiopathic Arthritis; UC, Ulcerative colitis; HS, Hidradenitis suppurativa; EpCAM, Epithelial cell adhesion molecule; HLA-DR, Human Leukocyte Antigen–DR isotype; HL, Hodgkin's lymphoma; HLA-DR, Human Leukocyte Antigen–DR isotype; MSLN, Mesothelin; NCI, The National Cancer Institute; PD-L1, Programmed cell death-ligand 1; NSCLC, Non-small cell lung cancer; SCLC, Small cell lung cancers; RCC, Renal cell carcinoma; HCC, Hepatocellular carcinoma; mMCC, metastatic Merkel cell carcinoma; BLyS, B-lymphocyte stimulator; SLE, Systemic Lupus Erythematosus; CCL11, CC chemokine ligand 11; ActRII, Myostatin/activin type II receptor; CCL2, CC chemokine ligand 2; MCP-1, Monocyte chemoattractant protein 1; IGF1R, Insulin-like growth factor 1 receptor; TGFβ, Transforming growth factor β; IL17A, Interleukin-17A; TRAIL-1, TNF-related apoptosis- inducing ligand receptor 1; NHL, Non-Hodgkin's lymphoma; GM-CSFRα, Granulocyte macrophage-colony stimulating factor receptor alpha; GCA, Giant cell arteritis; COVID-19, novel coronavirus 2019; HCL, Hairy cell leukemia; GM-CSF, Granulocyte macrophage-colony stimulating factor receptor; EGFR, Epidermal Growth Factor Receptor; LINGO-1, Leucine-rich repeat and Ig containing Nogo receptor interacting protein-1; MS, Multiple sclerosis; VEGFR2, Vascular endothelial growth factor receptor 2; Ang-1 and−2, Angiopoietin 1 and 2; VWF, von Willebrand factor; aTTP, Acquired thrombotic thrombocytopenic purpure; VEGFR2, Vascular endothelial growth factor-receptor 2; VEGFA, Vascular endothelial growth factor A; nAMD, Neovascular age-related macular degeneration; MEfRVO, Macular edema following Retinal Vein Occlusion; DME, Diabetic macular edema; CNV, Visual impairment due to choroidal neovascularisation; MM, Multiple myeloma; EDB-FN, Extradomain-B of fibronectin; IL2, Interleukin 2; PA, Protective antigen; OS, Osteoarthritis; HER3, Human epidermal growth factor receptor 3; BAFF-R, B-cell-activating factor receptor; CLL, Chronic lymphocytic leukemia; ETH, Swiss Federal Institute of Technology Zürich; pSS, Primary Sjögren's syndrome; AML, Acute myeloid leukemia; IGF-II, Insulin-like growth factor II; SOST, Sclerostin; OI, Osteogenesis imperfecta; HPP, Hypophosphatasia; BMD, Bone mineral density; VEGFR-3, Vascular endothelial growth factor receptor-3; FGFR2, Fibroblast growth factor receptor 2; TFPI, Tissue factor pathway inhibitor; FXI, Coagulation factor XI; VTE, Venous thromboembolism; COPD, Chronic obstructive pulmonary disease; TLR-3, Toll-Like Receptor 3; DKK1, Dickkopf 1; EDA-FN, Extra-domain A of fibronectin; HER2, Human epidermal growth factor receptor 2; LYPD3, Ly6/PLAUR domain-containing protein 3; pKal, Plasma kallikrein; HAE, Hereditary Angioedema, TRAIL-R2, TRAIL receptor 2; DR5, death receptor 5; SCLC, Small cell lung cancer; HER3, Human epidermal growth factor receptor 3; HNSCC, Head and neck squamous cell carcinoma; Aβ, Amyloid-β; FZD, Frizzled receptor; PNH, Paroxysmal nocturnal haemoglobinuria; AMD, Age-related macular degeneration; HLH, Hemophagocytic lymphohistiocytosis; MIF, Macrophage migration inhibitory factor; ICAM1, Intercellular adhesion molecule 1; oxLDL, Oxidized low-density lipoprotein; ChoP, Phosphorylcholine; MI, Myocardial infarction; NiV, Nipah virus; HeV, Hendra virus; HGFR, Hepatocyte growth factor receptor*.

The most dominant antibody format of the approved or under clinical investigations phage-derived antibodies is the Immunoglobulin G (IgG), yet other formats, such as antibody conjugates or nanobodies are also included (Table 2). MAbs isolated from the CAT libraries, for instance, belong to two IgG subclasses, IgG1 and IgG4, with the majority being IgG1-λ. MorphoSys's HuCAL® platforms show similar trend to CAT in addition to large number of mAbs from IgG2 subclass. MAbs from Dyax libraries, on the other hand, belong to IgG1 and IgG2 with the majority being IgG1-κ.

Having more than one mAb against a specific target or condition is essential especially that patients might acquire resistance against a prescribed therapeutic mAb, because of the possible immunogenicity and induction of anti-drug antibodies (ADAs) (459, 460). As a result, their pharmacokinetic, safety, and efficacy can be negatively impacted by the presence of ADAs (459–461). From this perspective, antibody phage display technology has enabled receptors like mesothelin (MSLN), human epidermal growth factor receptor 3 (HER3) and programmed cell death-ligand 1 (PD-L1) to have more than one specific therapeutic mAb (Table 2).

## Phage Display-Derived mAbs

As discussed earlier, phage display technology demonstrated its robustness and reproducibility as a human antibodies discovery platform. To date, 14 approved mAbs and many others in pre-clinical development or in clinical trials have been derived using this technology. These antibodies and antibody fragments are indicated to treat several disease conditions (Table 2). In this section, we will discuss in detail all the approved phage display-derived antibodies to highlight the utility of antibody phage display technology in the universe of biopharmaceutical industry.

## Atezolizumab (Tecentriq™)

Atezolizumab is a humanized IgG1-κ immune checkpoint inhibitor targets PD-L1 that commonly expressed on the surface of antigen presenting cells and tumor cells, and prevents its binding to the programmed cell death protein 1 (PD-1) receptor on T cells. PD-L1 is usually released by tumor cells and results in cancer immune evasion and decreases antitumor T-cell responses which are usually associated with poor clinical outcomes. Thus, utilizing atezolizumab could disrupt such T cell suppression by blocking PD-L1 binding to PD-1 and restore tumor-specific T-cell immunity in several cancer types (462–473).

In 2016, atezolizumab was approved by the US FDA for the treatment of urothelial carcinoma (UC) and metastatic lung cancer. Subsequently, it was granted accelerated approval for the treatment of advanced bladder cancer in 2017, and metastatic non-small-cell lung carcinoma (NSCLC) in combination with bevacizumab and chemotherapy in 2018. More recently, atezolizumab was approved for several indications, such as in combination with abraxane for patients with PD-L1-positive metastatic triple-negative breast cancer (PD-L1–positive TNBC), in combination with chemotherapy for the initial treatment of adults with extensive-stage small-cell lung carcinoma (SCLC), and in combination with abraxane and carboplatin for the initial treatment of metastatic non-squamous NSCLC (Table 2).

Clinical trials with atezolizumab are currently ongoing for multiple forms of solid tumors and hematologic malignancies. As of 2019, there are 249 ongoing trials with atezolizumab either as monotherapy or in combination with other anti-cancer agents. Ongoing clinical studies include several indications, such as NSCLC, UC, renal cell carcinoma (RCC), hepatocellular carcinoma, TNBC, colorectal cancer, and hematologic malignancies among other tumor types. Atezolizumab as a single treatment has shown a significant anti-tumor response in NSCLC (469, 474–476), UC (466, 477), glioblastoma multiforme (478), and RCC (479). In a randomized Phase II clinical trial for NSCLC, atezolizumab single treatment has shown an overall survival benefit compared to docetaxel (469).

## Avelumab (Bavencio®)

Avelumab is a fully human IgG1-λ immune checkpoint inhibitor that targets PD-L1 protein and blocks its interaction with PD-1. Additionally, avelumab is thought to engage the innate immune system and elicits an antibody-dependent cellular cytotoxic (ADCC) response against PD-L1-expressing tumors (480). While ADCC has not been indicated to contribute to the clinical activity of avelumab (472), preclinical studies suggest a possible role of ADCC in the activity of avelumab (481, 482).

In early 2017, Avelumab was approved for metastatic Merkel cell carcinoma (mMCC) in adults and pediatric patients aged >12 years as the first approved medication for this indication in the USA (483). In Europe, the application of avelumab marketing authorization for the treatment of mMCC is under regulatory review, while in Australia, Japan, and Switzerland phase II trial has been initiated for mMCC (483).

In 2017, the US FDA approved avelumab in the treatment of locally advanced or metastatic urothelial carcinoma metastatic urothelial carcinoma based on the phase III JAVELIN Bladder 100 trial (NCT02603432). Additionally, in 2019 the US FDA approved avelumab for the treatment of advanced RCC in combination with the tyrosine kinase inhibitor, axitinib. The approval was based on the phase III JAVELIN Renal 101 trial (NCT02684006).

Avelumab is under phase III trial in several countries for breast cancer, head and neck cancer, NSCLC, ovarian cancer, B cell lymphoma, and gastric cancer. There are many other phase II clinical trials underway globally for glioblastoma, intestinal cancer, nasopharyngeal cancer, endometrial cancer, recurrent respiratory papillomatosis, and thymoma (483).

## Moxetumomab Pasudotox-tdfk (Lumoxiti™)

Moxetumomab pasudotox-tdfk (CAT-8015) is a novel recombinant immunotoxin that consists of a recombinant murine scFv genetically fused to a truncated pseudomonas exotoxin (PE38), which targets CD22 antigen that is expressed on the surface of many types of malignant B cells including hairy cell leukemia (HCL) (263, 484). This mAb is the second generation of BL22/CAT-3888, whereby the CDRH3 has been affinity matured by phage display to increase the affinity by 14-fold toward CD22 (485, 486). After binding to CD22, moxetumomab pasudotox-tdfk is internalized, and the Pseudomonas exotoxin catalyzes inhibition of protein synthesis by ADP-ribosylation of elongation factor 2, resulting in apoptotic cell death (263).

HCL is a rare chronic disease that accounts for 2% of all leukemias with a 4:1 male-to-female predominance (487, 488). Outcomes with standard treatment are usually positive in 78% of patients however, relapses occur in ~50% of the patients (489). In 2018, the US FDA approved moxetumomab pasudotox-tdfk under the trade name of Lumoxiti™ (AstraZeneca Pharmaceuticals LP) to be utilized therapeutically for adult patients with relapsed or refractory HCL (R/R HCL) that no longer responding to other therapies, including purine analog (263, 490). Lumoxiti received US Orphan Drug designation and the FDA granted the application Fast Track and Priority Review designations because of the severity and rarity of the disease and was the first new therapy granted approval for HCL since cladribine in 1993.

Currently, the national cancer institute is sponsoring a phase I clinical trial to assess the safety of moxetumomab Pasudotox-tdfk in combination with rituximab in subjects with HCL or HCL variant (NCT03805932). Furthermore, an active phase III clinical trial (NCT04125290) aims to evaluate the post-marketing safety of moxetumomab pasudotox-tdfk for old patients (≥65 years), and/or patients with moderate renal impairment.

## Necitumumab (Portrazza™)

Necitumumab is a fully human IgG1-κ mAb which selectively binds the epidermal growth factor receptor (EGFR). It binds to domain III of the extracellular region of EGFR and blocks ligand binding. Necitumumab prevents the proliferation of several cancer cell lines by affecting downstream signaling of the EGFR receptor involved in cell growth and angiogenesis (270, 491) which are crucial for promoting growth and spread of cancerous cells. Specifically, it inhibits downstream signaling pathways, such as mitogen-activated protein kinase (MAPK) and phosphatidylinositol-4,5-bisphosphate 3-kinase (PI3k)/Akt activation which in turn inhibit cancer cell proliferation, differentiation, adhesion, migration and survival (492–494). EGFR overexpression has been found in about 40–80% of lung cancer patients as well as in many other cancers including squamous NSCLC (495, 496).

Necitumumab was firstly approved by US FDA for the treatment of metastatic squamous NSCLC combined with gemcitabine and cisplatin in 2015 (497). Clinical trials for necitumumab were initiated in 2004 (498), and currently, it is being tested in 6 clinical trials (NCT02496663, NCT00982111, NCT02789345, NCT00981058, NCT03944772, NCT03387111) mostly on NSCLC.

## Adalimumab (Humira®)

Adalimumab is the first phage display human IgG1-κ derived mAb developed by humanization with a “guided selection method” involving a mouse mAb (180). In 2002, adalimumab was the first human antibody derived from phage display that was granted approval for therapy by US FDA (Table 2) (172). Adalimumab is the biggest selling drug worldwide with $19.1 billion in 2019 and $82.5 billion cumulatively between 2014 and 2018 (7, 8). It shows very high specificity and sub-nanomolar affinity as it binds with tumor necrosis factor (TNF) and inhibits TNF receptors (TNF-R1 and -R2) binding and activation (499). This inhibition pathway leads to a wide range of anti-inflammatory responses as TNF is a key regulator for the initiation of proinflammatory cytokine cascade which ultimately leads to cell activation, inflammation, fever, and apoptosis (500).

Adalimumab was firstly indicated as a therapeutic option for some moderate and severe types of rheumatoid arthritis (RA) as monotherapy or in conjunction with MTX or other anti-rheumatic medications. Nowadays, adalimumab is one of the most prescribed medicines in immune-mediated disorders including RA (approved in 2002), psoriatic arthritis (PsA) (approved in 2005), ankylosing spondylitis (approved in 2006), Crohn's disease (approved in 2010), psoriasis and juvenile idiopathic arthritis (approved in 2008), ulcerative colitis (approved in 2012), hidradenitis suppurativa (HS) (approved in 2015), uveitis (approved in 2016), and fingernail psoriasis (approved in 2018). Currently, there are 118 clinical trials listed in ClinicalTrials.gov for adalimumab, and these studies ranging between phase I to phase IV.

## Raxibacumab (Abthrax®)

Raxibacumab is a human IgG1-λ human mAb that was produced from a naive human scFv phage display library licensed from Cambridge Antibody Technology (CAT) by Human Genome Sciences (HGS), which has been later acquired by GlaxoSmithKline (GSK) (331, 501). In 2012, raxibacumab was first granted FDA approval under the trade name of Abthrax® to be indicated as a prophylaxis for the treatment of inhalational anthrax in combination with some antibiotics. Anthrax infection is caused by bacteria called Bacillus anthracis (B. anthracis) through skin abrasions, inhalation or ingestion, where its spores are usually phagocytosed by macrophages (502). Moreover, *B. anthracis* is categorized as a potential biological weapon according to the US Centers for Disease Control and Prevention (CDC) (503).

*B. anthracis* secretes the lethal toxin (LT) and the edema toxin (ET). The LT is formed when the lethal factor (LF) interacts with the protective antigen (PA), which a cell-binding protein, while the ET is formed by an interaction between the PA and the edema factor (EF) (504). Raxibacumab targets the PA in *B. Anthracis* with high affinity to the LT (505) and acts through neutralizing PA with a nanomolar concentrations (IC_50_ is ~0.21 nm and Kd is ~2.78 nM). The mechanism of action of raxibacumab depends on the downregulation of the cellular uptake of toxins to prevent the development of lethal complexes (332).

Currently, in the US, raxibacumab is not only indicated for the prophylaxis of inhaled anthrax but also when alternative therapeutic options do not exist or are not suitable, such as treatment for an antibiotic-resistant strain of B. anthracis. Raxibacumab monotherapy of antibiotic-resistant *B. anthracis* infection suggests a benefit for up to 6 days post-exposure (NCT00639678) (506).

## Belimumab (Benlysta®)

Belimumab is a human IgG1-λ mAb that was discovered through a collaboration between HGS and CAT. Belimumab recognizes and binds to the soluble B lymphocyte stimulator (BLyS), preventing its interaction with its receptors (507). BLyS is a critical factor in the selection, maturation, and survival of B cells (508). BLyS is produced by a wide variety of cell types, including myeloid lineage cells, activated T cells, malignant B cells, and stromal cells (509–513). BLyS has three receptors that are expressed predominantly on B lineage cells, and some are found on subsets of activated T cells and dendritic cells (514, 515).

Patients with systemic lupus erythematosus (SLE) have elevated levels of BLyS, which correlate with high levels of autoantibodies and disease activity (516). Belimumab was FDA approved in 2011 and considered as the first biological drug, immunosuppressant, approved for the treatment of SLE. Long-term belimumab treatment causes a significant reduction of most plasma cells that are responsible for autoantibodies production (517, 518).

Belimumab is currently being tested in seven active clinical trials. These include a phase IV clinical trial to identify the side effects of belimumab when given with other SLE medications in adults with active SLE (NCT01705977). Also, in a Phase II study to evaluate the efficacy and safety of belimumab as a maintenance therapy in adults with refractory idiopathic inflammatory myositis (IIM) (NCT02347891). It is also being investigated in Phase II studies to evaluate its efficacy in combination with rituximab in adults with systemic SLE (NCT02426125) and in subjects with primary Sjogren's syndrome (NCT02631538).

## Ramucirumab (Cyramza®)

Ramucirumab is a fully human IgG1-κ mAb that inhibits vascular endothelial growth factor receptor-2 (VEGFR-2), thus inhibiting downstream signaling and preventing angiogenesis within tumors (519). In the adults, VEGFR-2 is predominantly expressed on vascular endothelial cells of blood vessels (520). Increased levels of VEGFR-2 have been detected in mammary, colorectal cancer, NSCLC, UC, and several other cancers (521).

The FDA approved ramucirumab in 2014 for use in the second-line setting as a single-agent treatment for advanced or metastatic gastric cancer or gastroesophageal junction adenocarcinoma (522). Also, in 2014, ramucirumab was approved in combination with docetaxel, for treatment of metastatic NSCLC (523). In 2015, it was approved for use with FOLFIRI as a second-line treatment of metastatic colorectal cancer (524). In 2019, ramucirumab became the first FDA-approved biomarker-driven therapy in patients with hepatocellular carcinoma for people who have high levels of alpha-fetoprotein (525).

Currently, ramucirumab is being investigated in 19 different clinical trials for several other indications including a phase II randomized trial in combination with mFOLFIRINOX in patients with advanced pancreatic cancer (NCT02581215), phase III study in combination with chemotherapy treatment in previously untreated patients with HER2-negative, unresectable, locally-recurrent, or metastatic breast cancer (NCT00703326), and phase III trial in combination with docetaxel in patients with locally advanced or unresectable or metastatic UC (NCT02426125).

## Guselkumab (Tremfya™)

Guselkumab is a human IgG1-λ mAb that neutralizes interleukin-23 (IL-23) functions (526). IL-23 is a pleiotropic, heterodimeric cytokine, consisting of a p19 and a p40 subunits, which are primarily secreted by antigen presenting cells, such as macrophages and dendritic cells (527). IL-23 belongs to the IL-6/IL-12 family of cytokines that have a crucial role in numerous immune responses (528). IL-23 specifically induces Th-17 proliferation and the subsequent release of IL-17 cytokine, which triggers inflammatory and autoimmune disorders, such as psoriasis (529). Guselkumab binds to IL-23p19 subunit with high affinity and specificity, inhibiting interaction with its receptor on the cell surface of certain immune cell subsets, most importantly on Th17 cells (529, 530). Such activity is responsible for preventing the activation of the IL-23 receptor and the subsequent production of several proinflammatory cytokines.

Guselkumab has demonstrated safety and efficacy in several clinical trials including a phase II proof-of-concept trial, which demonstrated efficacy in all endpoints linked to health-related quality of life as well as joint signs and symptoms, and skin disease (399, 400, 531). Patients from this study have also experienced a dramatic decrease in IL-17A, IL-17F, and C-reactive protein in their serum to normal levels compared with healthy controls, highlighting the significance of suppressing the IL-23/Th17 pathway for the treatment of skin and joint disorders (531). This encouraging trial has led to two pivotal phase III clinical trials, DISCOVER-1 (NCT03162796) and DISCOVER-2 (NCT03162796), where patients had experienced an improved joint, skin, physical function and health-related quality of life (532, 533). In 2017, this mAb has received approval from the US FDA to treat adults with moderate to severe plaque psoriasis who are candidates for systemic therapy or phototherapy (398) and is currently being evaluated in six active clinical trials at different phases. These include the evaluation of efficacy, safety and tolerability in patients with moderate to severe HS (NCT03628924) as well as patients with chronic plaque-type psoriasis refractory to ustekinumab treatment (NCT03553823).

## Lanadelumab (Takhzyro®)

Lanadelumab is a fully human IgG1-κ mAb that inhibits the proteolytic activity of plasma kallikrein (PK) enzyme. PK enzyme induces the proteolysis of the coagulation factor XII and prekallikrein (pKal), and a non-enzymatic high-molecular-weight kininogen (HMWK) to generate bradykinin in response to tissue injury and pathophysiological stimuli (534–536). The increased level of bradykinin leads to angioedema episodes, an allergic skin swelling condition, through its excessive vasodilation effect (537, 538). This syndrome is a clinical feature of patients with hereditary angioedema (HAE), in which, a mutation in the *SERPING1* gene leads to a reduced expression of C1 protein that lessens its function as a bradykinin regulator (539). Furthermore, certain mutations in the *F12* gene result in the production of factor XII with increased activity leading to excessive production of bradykinin (540).

In phase I clinical trial, lanadelumab demonstrated a favorable safety profile with a potential inhibitory effect on HMWK and a long-term prevention of HAE attacks, enabling further evaluation in a larger trial (541, 542). Accordingly, a phase III randomized clinical trial (NCT02586805) evaluated the efficacy and safety of lanadelumab to prevent HAE attacks in patients with symptomatic HAE due to C1 inhibitor deficiency (C1-INH-HAE) disorder (403). In this trial, 150 or 300 mg were evaluated in subcutaneous injections setting, given every 2–4 weeks over a 6-months period. The findings from this study have demonstrated the efficacy of lanadelumab in preventing HAE attacks, leading to its approval by the US FDA in 2018 for the treatment of patients with type I or II HAE. In 2018, lanadelumab has received the US FDA approval for the prevention of the angioedema attacks in patients with hereditary angioedema. Currently, it is being evaluated in two active phase III clinical trials to prevent hereditary angioedema attacks in pediatric patients as well as in adolescent and adult patients suffering from acquired angioedema (AAE) due to C1-INH deficiency (NCT04070326 and NCT04206605).

## Ixekizumab (Taltz®)

Ixekizumab is a humanized IgG4-κ mAb that targets IL-17A cytokine, which is a member of IL-17 cytokines family mainly produced by CD4-Th17 cells. Several other immune cells residing in the gut, lung and skin, including a subset of natural killer (NK) cells, Paneth cells and neutrophils also produce IL-17A in response to IL-23 cytokine stimulation (248, 543). The main function of Th17 cells is to clear pathogens not properly handled by the Th1 or Th2 immune response (544, 545). The infiltration of Th17 cells under the skin and excessive production of IL17A lead to the pathophysiology of psoriasis and PsA (546). The latter is an inflammatory disease with articular, peri-articular and extra-articular features that leads to skin and joint damage, and loss of functions (547). Several attributes lead to this condition including immunologic, genetic and environmental factors where a combination of two or more of these factors trigger the inflammatory immune response (548–551). There has been growing evidence suggesting the involvement of IL-17 signaling in PsA pathogenesis (552), which involves a persistent activation of Th-17 cells in response to synovial or skin antigens leading to tissue destruction and joint remodeling (552).

Several randomized clinical trials have assessed the efficacy of ixekizumab in patients with PsA achieving a primary endpoint of American College of Rheumatology 20% improvement (ACR20) (NCT01624233, NCT02349295, NCT01695239, NCT02584855). Moreover, ixekizumab has been proven to be superior to conventional rheumatic drugs as well as TNF-α inhibitors in two phase III clinical trials, indicating its safety and efficacy in delaying disease progression and supporting its use as a front-line therapy for PsA (249, 553, 554).

Lxekizumab was initially approved in 2016 by the US FDA for moderate to severe plaque psoriasis treatment in adult patients who are eligible for systematic therapy or phototherapy. The FDA approval was further expanded in 2017 for the treatment of adults suffering from active PsA. Furthermore, the US FDA approved Ixekizumab in 2019 for the treatment of active ankylosing spondylitis in adults. In early 2020, Ixekizumab has also granted the US FDA approval for the treatment of pediatric patients (ages 6 to 18) with moderate to severe plaque psoriasis who are also eligible for systemic therapy or phototherapy. Currently, ixekizumab is being evaluated in four active clinical trials. These include a phase IV clinical trial assessing the efficacy of ixekizumab in Japanese patients with generalized pustular psoriasis and erythrodermic psoriasis (NCT03942042). In addition, this mAb is also being tested in Chinese patients who have moderate to severe plaque psoriasis (NCT03364309).

## Ranibizumab (Lucentis®)

Ranibizumab is a Fab antibody fragment derived from a recombinant humanized IgG1-κ mAb (murine Mab A.4.6.1) (555). Ranibizumab was created from the same parent mouse antibody as bevacizumab to target VEGF-A, both bind effectively and neutralize VEGF-A isoforms (555). VEGF-A and its receptors VEGFR-1 and VEGFR-2 promote angiogenesis throughout the body and represent the primary mediators of degenerative ocular conditions, such as diabetic retinopathy, retinal vein occlusions, age-related macular degeneration (AMD) including wet-AMD, the leading cause of blindness in the elderly population (556, 557). Ranibizumab has smaller size than bevacizumab and readily penetrates all layers of the retina after intravitreal injection (558). Importantly, ranibizumab is thought to be safer on normal healthy cells that express VEGF-A as it has shorter serum half-life and faster system clearance (559). The US FDA approved ranibizumab for the treatment of wet AMD in June 2006, macular edema following retinal vein occlusion in 2010, diabetic macular edema in 2012, diabetic retinopathy in people with diabetic macular edema in 2015, and myopic choroidal neovascularization in 2017.

Currently, there are 18 active clinical trials to evaluate ranibizumab as single or in combination with other treatments. One phase IV clinical study is comparing the safety and efficacy between ranibizumab as monotherapy and in combination with R:GEN (selective retina therapy) in clinically significant diabetic macular edema (NCT03759860). Also, another phase IV clinical study is comparing intravitreal ranibizumab and triamcinolone acetonide combination therapy vs. ranibizumab monotherapy in patients with polypoidal choroidal vasculopathy (NCT02806752). Long-term efficacy and safety of intravitreal ranibizumab compared with laser ablation therapy in patients who were treated for retinopathy of prematurity (ROP) (NCT02640664) is in phase III stage.

## Caplacizumab (Cablivi™)

Caplacizumab is a humanized bivalent single-variable domain immunoglobulin (VHH) that consists of two identical, genetically linked, humanized building blocks (298). Caplacizumab was generated for the treatment of platelet adhesion diseases, such as acquired thrombotic thrombocytopenic purpura (aTTP) (298). Caplacizumab binds specifically to human von Willebrand factor (vWF) A1 domain, preventing its binding to the platelet glycoprotein 1b receptor (298). Acquired TTP is a rare blood disorder characterized by thrombosis in small blood vessels and low platelet count. It is caused by a severe deficiency in the vWF-cleaving protease (ADAMTS13) primarily due to acquired autoantibodies (560). Lacking ADAMTS13 enhances the accumulation of large multimers of vWF, vWF–platelet aggregation, and microvascular thrombosis of TTP, leading to low platelet count, ischemia, and organ dysfunction (560, 561).

Clinical studies showed that treatment with caplacizumab transiently reduced vWF levels and normalized platelet count compared with placebo (562). Target-bound caplacizumab is thought to be metabolized within the liver, while unbound caplacizumab is cleared renally (299). Caplacizumab received its first approval in September 2018 in the EU for the treatment of adults experiencing aTTP in conjunction with plasma exchange and immunosuppression (corticosteroids and increasingly also rituximab) (11). Caplacizumab is the first nanobody-based medication to receive approval in the US in 2019, for patients aged ≥18 years experiencing aTTP (11). Currently, one active phase III clinical trial (NCT02878603) is evaluating the long-term safety and efficacy of repeated use of caplacizumab in aTTP patients.

## Emapalumab (Gamifant®)

Emapalumab is a human IgG1-λ mAb that neutralizes interferon gamma (IFNγ) activities and inhibits its binding to the interferon receptors (IFNγR1 and IFNγR2). It binds with high affinity to free and receptor-bound IFNγ, preventing the downstream signaling of JAK/STAT pathway and the subsequent cytokine storm release (563, 564). This blockade results in the attenuation of the adaptive and innate immune responses, which increase the susceptibility to infections (564). It has been primarily developed to treat patients with haemophagocytic lymphohistiocytosis (HLH) disorder refractory to conventional therapy (565, 566). HLH is a rare pathologic immune activation syndrome with excessive inflammation that occurs as a familial or sporadic disorder due to a variety of immune triggers (567).

A phase I dose-escalation clinical trial investigating the safety in healthy subjects, revealed a favorable safety profile warranting further clinical development (NCT01459562). Emapalumab was then evaluated in an open-label phase II/III trial in 34 pediatric patients with a confirmed HLH disorder. Emapalumab was administered with dexamethasone intravenously every 3–4 days for a minimum of 4 weeks and up to 8 weeks with a primary endpoint of overall response rate (ORR) (NCT01818492). Patients have experienced a response rates above 70 with a safe and tolerable profile and mild-moderate infusion-related reactions in 27% of the patients confirming its favorable outcomes (568). This trial has led to the US FDA approval of emapalumab in 2018 as an IFN blocking molecule for pediatric and adult patients with HLH refractory to conventional therapy (dexamethasone, etoposide, and intrathecal methotrexate) (565, 566). Currently, the efficacy of emapalumab is being evaluated in four active phase II/III clinical trials in patients with primary and secondary HLH (NCT03312751, NCT03985423, NCT01818492, NCT03311854). In addition, the safety and efficacy of emapalumab are also being tested in COVID-19 patients, with a primary endpoint of reducing the number of patients who need invasive mechanical ventilation or extracorporeal membrane oxygenation (NCT04324021).

## Conclusion and Future Prospects

Antibody phage display is a versatile, reproducible, and functional technology that can be utilized to isolate antibody candidates for numerous disease indications. While it is the most common and well-established form of display technologies, the success of isolating useful antibodies is highly dependent on the quality and the nature of the targeted antigen used in biopanning and the size and quality of the library. By performing cell-based biopanning, antibody phage display can be used to identify new cell biomarkers, isolate antibodies that can discriminate between different antigen epitopes and conformations, or identify antibodies against antigens that are not available in pure form (74).

Phage display technology has been proven to be a powerful human mAb discovery platform. Not long ago the commercial use of phage display was restricted to a few selected biopharmaceutical companies with rights to phage display intellectual property (569). This explains why most of the approved mAbs or those in clinical trials sourced from phage display libraries belong to commercial entities with rights to the technology. However, most of the key patents covering phage display technology have expired in Europe and the US (569). Consequently, patent expiration should encourage academic and biotech start-ups to develop their own libraries to generate and develop more antibodies for translation to the clinic.

By reflecting on the collected data, antibody phage display has contributed to the isolation of antibodies for the treatment of many disease indications. There are 14 FDA approved phage display-derived antibodies and antibody fragments, and many others in clinical trials. Many research institutes, start-ups and industrial laboratories are continually developing methods for the design, construction and screening of developable antibody-phage libraries. Further improvements are expected to be achieved in the near future as this technology contributes significantly toward research, diagnosis, and therapy.

## Author Contributions

MA and AH designed the review. MA, HA, AM, AA, and MJ wrote the main manuscript text. MA prepared the figures and analyzed the data. MJ, SM, and AH provided a substantial contribution to the discussion of the content and helped with reviewing and editing the article. All authors read and approved the final manuscript.

## Conflict of Interest

The authors declare that the research was conducted in the absence of any commercial or financial relationships that could be construed as a potential conflict of interest.
